# Perceptions of peer contraceptive use and its influence on contraceptive method use and choice among young women and men in Kenya: a quantitative cross-sectional study

**DOI:** 10.1186/s12978-022-01331-y

**Published:** 2022-01-21

**Authors:** Lisa M. Calhoun, Anastasia Mirzoyants, Sylvia Thuku, Lenka Benova, Therese Delvaux, Thomas van den Akker, Courtney McGuire, Bernard Onyango, Ilene S. Speizer

**Affiliations:** 1 Carolina Population Center, The University of North Carolina at Chapel Hill, Chapel Hill, NC USA; 2 Athena Institute, Vrije Universiteit, Amsterdam, The Netherlands; 3 Shujaaz Inc., Nairobi, Kenya; 4 Department of Public Health, Institute of Tropical Medicine, Antwerp, Belgium; 5 Department of Obstetrics and Gynaecology, Leiden University Medical Center, Leiden, The Netherlands; 6 The African Institute for Development Policy, Nairobi, Kenya; 7 Department of Maternal and Child Health, Gillings School of Global Public Health, The University of North Carolina at Chapel Hill, Chapel Hill, NC USA

**Keywords:** Contraceptives, Family planning, Youth, Social norms, Peer, Condom, Kenya

## Abstract

**Background:**

Prior research has established that an individual’s social environment may influence his or her reproductive behaviors, yet less is known about peer influence on contraceptive use among young people (ages 15–24). In Kenya, the site of this study, 15% of adolescents ages 15–19 have begun childbearing and 45% of sexually active young women report current use of a modern contraceptive method. This highlights the need to better understand what factors influence young people to use contraception. The objective of this study is to explore the relationship between the perception of peers’ use of contraceptives and contraceptive use and method choice among young men and women in Kenya.

**Methods:**

This study utilizes a nationally representative sample of women and men aged 15–24 years from the 2018 and 2019 cross sectional Shujaaz State of the Kenyan Youth annual surveys. Among the sample of sexually experienced young people (59%), multivariable multinomial logistic regression was used to explore the association between the perception of peers’ use of contraceptives and the respondent’s contraceptive method choice: non-user, condom use or use of any other modern method. Results are presented separately for young men and young women.

**Results:**

Our results show that sexually experienced young men and women who perceive that their peers are using contraceptives are more likely to report current use of condoms compared to being a nonuser (RRR = 2.12, p < 0.001, RRR = 2.59, p < 0.001, respectively); they are also more likely to use condoms than another modern method of contraception (RRR = 2.13, p = 0.034, RRR = 1.71, p = 0.014, respectively). Young women are more likely to use another modern method (not including condoms) than be a nonuser when they perceive that their peers’ use contraceptives (RRR = 1.51, p = 0.020).

**Conclusions:**

The results of this study highlight the important role of peer influence on young people’s contraceptive choices. These findings can be used to develop programs that encourage behavior change communication activities in Kenya that focus on normalizing use of a full range of contraceptive methods among peer groups of sexually experienced young people.

## Plain language summary

In Kenya, about 15% of women age 15–19 have already had a birth and approximately one third of these women did not want to have a child at that time. Yet about 45% of sexually active women ages 15–24 report that they currently use family planning (FP). Among young people, friends and peers are an important influence on behaviors.

This study focused on a representative sample from Kenya of female and male youth (ages 15–24) who ever had sex. Men and women were asked questions about use of FP, if they think their peers use FP and about characteristics such as age and education.

The results showed that young women and men who believed their peers use FP were more likely to use FP themselves. Also, young men and women who believed that peers use FP were more likely to use condoms than not use any FP and more likely to use condoms than to use another modern method of FP. Young women who thought their peers use were more likely to use another modern method (not including condoms) than to be a nonuser of FP.

Programs targeting young people should include information on a range of FP methods and aim to include groups of peers and encourage open discussion.

## Background

Family planning (FP) is widely recognized for its role in reducing maternal and infant mortality as well as enabling women, men and couples to choose when and if they would like to have children [1]. Yet, about 218 million women of reproductive age in low- and middle- income countries (LMIC) report that they would like to avoid a pregnancy but are not using a modern family planning method, a concept commonly referred to as unmet need for modern contraception [1]. Unmet need is high among young people in sub-Saharan Africa (SSA) and as a result, approximately 37% of pregnancies among adolescent girls ages 15–19 and 27% of pregnancies among women ages 20–24 in SSA are unintended [1, 2]. To address these issues, the global community has recognized the importance of ensuring that the sexual and reproductive health needs of young people are met through enabling access to high quality family planning care for all [3–6]. Given that young people often experience many key life transitions between ages 15–24, such as initiation of sexual activity, marriage and childbearing [7–9], it is critical that they have access to information and services to meet their evolving reproductive health needs in this period [3–5].

Kenya, the site of this study, has been the focus of international attention in recent years due to concerns about high levels of teenage pregnancy, recently considered to be exacerbated by the global COVID-19 pandemic [10, 11]. The 2014 Kenya Demographic Health Survey (KDHS) shows that 15% of young women ages 15–19 have already begun childbearing and 3% were pregnant with their first child [12]. Kenyan women and men typically have their first sexual experience in their teenage years and women’s median age for first birth is 20.3 years [12]. These overall estimates mask differences by region, wealth status and education level, with sexual initiation and age at first birth occurring earlier for young people living in rural areas, of lower wealth status and with less education [7–9, 12]. Additionally, the adolescent and youth years are characterized by evolving family planning needs depending on age, relationship status, life goals and fertility desires [13, 14]. Modern contraceptive use in Kenya among young women ages 15–24 who are in union is almost 10 percentage points higher than that of unmarried sexually active young women (56% vs. 48%) [9]. Further, contraceptive method mix among young women in union is dominated by injectables and implants whereas unmarried, sexually active young women use a broader set of methods with higher use of condoms as compared to their married counterparts [9]. Contraceptive use and method mix are often not reported for men, though some studies have shown that young men report using predominantly condoms for dual prevention of pregnancy and sexually transmitted infections [15, 16].

It is well established, both globally and in Kenya, that a woman’s social environment, inclusive of her family, peers and community, plays an important role in influencing reproductive behaviors [17–22]. An individual’s behavior can be influenced through interactions with her or his social network, whereby broader social networks can serve as a source of new information and ideas and have been shown to be associated with contraceptive use [21, 23, 24]. Additionally, behavior can be influenced by social norms which guide social conduct and dictate what individuals should and should not do [25]. There is growing interest in exploring the role of social norms on FP behaviors, including modern contraceptive use [26–30]. In a review by Costenblader and colleagues (2017), the authors demonstrated that in all 17 included studies, which spanned the globe, there was a significant relationship between social norms supportive of contraceptive use and increased contraceptive use [26]. Yet few studies examined the role of social influences on contraceptive method choice, that is, not just the decision to use or not to use a method, but also the choice of method type. A study undertaken in Thailand in 1994 found that women with a more extended social network, defined as having more extended kinship ties, were more likely to use modern contraceptives and further, predicted use of oral pills, intrauterine device (IUD) and injectables increased as the number of kinship ties increased [24]. Using social network analysis, studies in Cameroon [31] and Bangladesh [32] found that there was a relationship between the method used by the respondent and the methods used by members of her social network, in that women were frequently connected to others who used similar methods. These studies reflect that a woman’s own contraceptive method choice is sensitive to the methods that her network members use.

The global community has long recognized the importance of including men in programs and research on FP, in part because spouses or partners are important decision makers regarding reproductive behaviors. Men have been shown to positively and negatively influence contraceptive use through couple communication, procurement of contraceptive methods, reinforcement of myths and stereotypes about contraceptive users and contraceptive methods and even restricting their partner from using contraceptives [33–36]. Qualitative research from rural Malawi shows that social influences function differently for men and women, with men making conclusions about contraceptive use in their community based on their own observations whereas women relied on direct conversations with their social network to inform their conclusions [37]. Further, a longitudinal study from rural Kenya has shown that men’s social networks may be more influential on men’s contraceptive use decisions as compared to the influence of women’s social networks on their contraceptive use [22]. This points to the need for more research on social influences on contraceptive use and choice among men.

Overall, the role of social influences on contraceptive use and method choice is understudied among young people in sub-Saharan Africa. This gap is notable given that adolescence and young adulthood is the time when social influences, particularly peers, are important and influential [38, 39]. Peers may play both a positive and negative role in influencing behaviors, as they have been shown to be a trusted source of information about family planning and at times model positive behaviors, but yet have also been found to share incorrect information, perpetuate myths and reinforce social norms that dictate when and if young people, particularly young women, should engage in sexual activity [33, 35, 40, 41]. Existing evidence among young people supports that peers influence contraceptive use [33, 35] and specifically condom use [42–45], but given that Kenya’s contraceptive method mix among young people includes a range of methods, including implants, injectables and condoms, it is important to better understand if social influences are associated with method use and choice, including commonly used hormonal methods. Access to hormonal methods for young people is beneficial in that hormonal methods can provide longer term protection from pregnancy, are more reliable, may be more cost effective, and are more convenient as compared to coitally-dependent methods such as condoms or emergency contraception; however, barriers to use of hormonal methods are common, including the belief that contraceptives cause infertility as well as fear of real side effects [46]. In this context of high teenage pregnancy and substantial evidence on the role of social influences on contraceptive use among women of reproductive age, it is important to understand the role of social influences on contraceptive method choice among both young women and men. This knowledge can inform programmatic strategies seeking to provide full, accurate information to young people and their peers to support young people’s use of the method of their choice.

This paper aims to address these gaps by utilizing data from young women and men ages 15–24 years in Kenya. Using cross-sectional data collected in 2018 and 2019, this paper explores the influence of perceptions of peers’ use of contraceptives on contraceptive method use and type of contraceptive method used among sexually experienced young women and men.

## Methods

### Data

The data for this paper come from consolidating the 2018 and 2019 cross-sectional, nationally representative Shujaaz State of the Kenyan Youth annual surveys. Shujaaz, Inc. (formerly Well Told Story) is an East African network of social ventures, whose mission is to deliver social and economic value to youth by producing insight-driven experience, consistent positive influence, and information that result in large scale social and behavior change among the Shujaaz, Inc. target audience. One of its ventures, Shujaaz media, is comprised of a monthly comic, daily engagements through digital channels, and events; all media are free to the audience. As part of routine audience research and program monitoring and evaluation, Shujaaz, Inc. undertakes collection of big data, qualitative and quantitative studies, including an annual household survey of young women and men in Kenya. These surveys include males and females ages 15–24 years. In each round, a new sample of female and male youth was selected. For the data used in this paper, a multi-stage sampling procedure was used to achieve the nationally representative sample of youth aged 15–24 years for each round. First, all counties in Kenya were divided into two strata—urban and rural. Then, target districts were selected within each stratum using a probability proportional to population size approach. The same approach was used to select enumeration areas (EA) within each selected district for a total of 202 EAs. In each selected EA, a household listing was undertaken with the head of household to identify all eligible households. Households were eligible if they had at least one member age 15–24. Ten households were selected in each EA using the random walk from a landmark chosen with the help of a Kish Grid. When there were multiple 15–24 year olds in the household, only one youth was selected using a Kish Grid. If the selected respondent was not at home at the time of the interview, an interviewer would make up to 3 call backs and then replace the respondent or the household using a predefined formula.

Upon giving consent for participation at the time of the survey (parental/guardian consent and adolescent assent in the case of minors aged 15–17, regardless of marital status), respondents were asked about sociodemographic characteristics, use and access to media, family planning use, agriculture activities and tobacco use. Study questionnaires were designed and pre-tested by the Shujaaz team which was comprised of young women and men ages 18–24. The survey questions were based on standardized surveys (e.g., DHS or PMA2020) and previous qualitative research by Shujaaz, Inc. and used a combination of youth-defined terms (including Sheng, a youth dialect), and asked questions in a way that resonated with young people, including several open-ended questions that aimed to build rapport between the interviewer and respondent. Before the first round of the survey, the data collection agency conducted cognitive interviews with young men and women in urban and rural areas, and also piloted the questionnaire prior to each survey round. The sample size in 2018 was 2020 (1009 males and 1011 females) and 2020 in 2019 (1023 males and 997 females). The datasets from 2018 and 2019 were combined in order to increase the sample size for analysis purposes.

### Population

The main outcome of interest is contraceptive use and method choice among sexually experienced young men and women aged 15–24 years. This analysis was limited to the 59% of young women (n = 1191) and 63% of young men (n = 1279) who reported that they ever had sex by the time of survey.

### Dependent variable

The dependent variable for this analysis is current contraceptive use and choice. Young men and women were asked if they had ever tried any contraceptive method in any relationship, which includes sexual encounters, dating relationships and marriage. If so, the interviewer listed each type of contraceptive method and asked the respondent to say if they had ever tried the contraceptive method or not for each method on the list. Those who had ever tried a method were asked to describe their current use of that method. Response options included “I use this in all or almost all sexual encounters, I always have it with me”, “I use it occasionally when I happen to have it with me”, “I use it occasionally, mostly when a partner has it with them”, “I use it as a back-up when another preventive method fails”, “I never use it”, “Other”, “Don’t know/Refused”. We coded respondents as current user of a method if they indicated that they used this method “in all or almost all sexual encounters, I always have it with me;” all other responses were considered to be inconsistent use and thus not reflecting ‘current use’. The small number of respondents who reported concurrent, consistent use of more than one method (n = 45) were coded as using whichever method was the most effective; the majority of the respondents who were concurrently using more than one method used a male condom and a hormonal method (n = 28 or 1.13% of the analysis sample). A categorical contraceptive use variable was created separately for males and females. Respondents who reported using traditional methods or non-use were coded as ‘0’ or “non-users of modern contraceptives”; those who used male condoms were coded ‘1’; those who reported that they or their partner used implants, intrauterine devices (coil/IUD), injectables, oral pills, female condoms and emergency contraceptives were coded ‘2’ as “All (or any) other modern method users”. We tested a categorical contraceptive use variable for young women which separated “all other modern method users” into those using a long-acting reversible method and those using a short-acting hormonal method. After further analysis it was determined that the results were more stable without this disaggregation.

### Independent variable

The questionnaire was designed to examine respondents’ perceptions of a descriptive norm: the perception of peer contraceptive use. This variable was based on two questions: (1) “How many of your friends use contraception to protect from pregnancy/sexually transmitted infections (STIs)?” and (2) “How many people your age, who are not your friends, use contraceptives to protect from pregnancy/STIs?”. Separate variables for friends and peers were created with the response options “All” and “Most” coded ‘1’ and “Some”, “None”, and “Don’t know/refused” coded ‘0’. The separate variables for friends and peers were found to be correlated at 0.59, and therefore, a combined measure was created which we refer to as ‘perceptions of peers’ use of FP’. If the respondent answered “All” or “Most” to either of the separate questions, the key independent variable was coded ‘1’. All others were coded ‘0’.

### Analysis

Descriptive statistics (proportions and means) were used to better understand differences between perceptions of peers’ use of FP and contraceptive method use and choice. Statistically significant differences were assessed using the Pearson Chi-square tests and t-tests to compare continuous variables.

Multinomial logistic regression models explored the influence of the key independent variable on current contraceptive use and choice separately among sexually experienced young women and men. We run the multinomial logistic regression models using two different referent groups in order to permit the exploration of all comparisons in the outcome variable. The results are presented as relative risk ratios. Multivariate analyses were run with Huber-White-adjusted standard errors to account for the clustering in the sample at the EA level and also adjust for demographic characteristics. Based on previous evidence demonstrating the relationship between demographic factors and contraceptive use [47, 48], the following variables were included as covariates in the multivariate analyses: age in years (continuous); education (none/some primary, primary completion, secondary completion, college or vocational school); number of children (none, one or more children) and residence status (urban, rural). Relationship status at the time of survey was also included as a covariate with the following categories: dating, have a boyfriend or girlfriend; single, do not have a boyfriend or girlfriend; and ever married/in union. A total of 17 young men and 17 young women reported that they were widowed or divorced; these 34 respondents were coded as “ever married” along with the respondents who were currently married. Additionally, the respondent’s reported average monthly earnings in Kenyan shillings (KES) was also included as a covariate (2018–2019 conversion rate of 1 KES is approximately US$0.00980). We also adjusted for survey wave (2018, 2019) to control for secular time trends. Despite the decision to include the covariates as a priori confounders, we checked multicollinearity across independent variables in both multivariate models and did not find evidence of multicollinearity. In both models, all variance inflation factors were less than five which suggests that there is some moderate correlation between covariates but not substantial enough to require changes to the model. In both models, the values for tolerance were all 0.2 or greater. In addition, interaction terms between the relationship status dummies and the peer influence exposure variable were created to explore if there were differences in peer influence by relationship status. The interaction terms were not significant and therefore are not presented. All analyses were performed using Stata version 16.

### Ethics

On behalf of Shujaaz Inc, Research Guide Africa, the subcontractor for data collection, obtained all required study permits from the National Commission for Science, Technology and Innovation (NACOSTI). This secondary analysis study was assessed by the University of North Carolina Institutional Review Board and determined exempt from further review (Study #21-0593).

## Results

Table 1 presents the characteristics of sexually experienced young women and men ages 15–24 who participated in the 2018 and 2019 Shujaaz State of the Kenyan Youth annual surveys. Young women and men had similar levels of education, average age at the time of survey, and residence status. A higher percentage of young men reported that they had a girlfriend at the time of the survey as compared to young women reporting to have a boyfriend (59.0% vs. 38.8%) whereas a higher percentage of young women reported being ever married as compared to young men (35.5% vs. 8.8%). A higher percentage of young women than men had one or more children. Current contraceptive use and choice patterns were different for young women and men, though there were similar percentages of non-users of modern methods among both women and men (57–59%). About 15% of young women reported current use of the male condom and about one quarter reported current use of any other modern method. Conversely, about 40% of young men reported using condoms and only 3% reported that their partner currently used another modern method.

**Table 1:** Descriptive characteristics of sexually experienced young men and women ages 15–24 surveyed in 2018 and 2019, Kenya

	Young men (n = 1279)	Young women (n = 1191)	Total (N = 2470)
Age in years (mean (median))	20.37 (20)	20.63 (21)	20.50 (21)
Education (%)			
None/some primary	12.3	15.7	13.9
Primary completion	38.7	41.7	40.2
Secondary completion	35.6	32.3	34.0
College or vocational training	13.4	10.3	11.9
Relationship status at time of survey (%)			
Single, do not have a boyfriend or girlfriend	32.2	25.7	29.1
Dating, have a boyfriend or girlfriend	59.0	38.8	49.3
Ever married or in union	8.8	35.5	21.6
Number of children (%)			
None	89.9	51.3	71.3
One or more	10.1	48.7	28.7
Reported average monthly earnings in KES (mean (median))	6939.33 (4000)	4437.14 (2500)	5732.81 (3000)
Residence status			
Rural	67.1	65.9	66.5
Urban	32.9	34.1	33.5
Survey wave			
2018	54.7	52.3	53.6
2019	45.3	47.7	46.4
Current contraceptive use and choice^a^			
Non-user of a modern method	56.5	58.9	57.7
Male condoms	40.4	15.8	28.5
All other modern methods^b^	3.1	25.3	13.8

^a^Current contraceptive use is based on the respondent’s current use of the most effective method mentioned. Only ~ 1% of the sample report dual use of a hormonal method and a condom. Our measure of current contraceptive use does not capture periodic use of a secondary method

^b^This category includes current users of implants, IUDs, injectables, oral pills, emergency contraceptive and the female condom

Table 2 presents the cross tabulation of perceptions of peers’ use of FP by current contraceptive use and method choice among sexually experienced young men and women. About 42% of young women and men reported that they thought all or most of their peers use FP to avoid pregnancy or protect from STI. Among male users of condoms, 54.2% believed all or most of their peers use FP whereas only 35.9% of users of any other modern method and 34.9% of nonusers perceived that all or most of their peers use FP. Among young women, 57.5% of condom users, 50.0% of other modern method users, and 54.2% of nonusers perceived that all or most of their peers use FP. Based on Pearson Chi-square tests, we found a significant association (p < 0.001) between perceptions of peers’ FP use and current contraceptive use and choice among young men and women.

**Table 2:** Perception of peers’ use of FP by current contraceptive use and choice^a^ among sexually experienced young men and women ages 15–24 in 2018 and 2019, Kenya

	Young men (n = 1279)				Young women (n = 1191)			
	Non-use/traditional method	Male condom	All other modern methods^b^	Total (%)	Non-use/traditional method	Male condom	All other modern methods^b^	Total (%)
Total (n)	723	517	39		517	188	302	
How many of your peers use contraception to protect from pregnancy or STI?								
All/Most	34.9	54.2	35.9	42.7	54.2	57.5	50.0	42.3
Some/none/don’t know	65.1	45.8	64.1	57.3	45.8	42.5	50.0	57.7
	100%	100%	100%	100%	100%	100%	100%	100%
	χ^2^ = 46.67, p ≤ 0.000				χ^2^ = 40.52, p ≤ 0.000			

^a^Current contraceptive use is based on the respondent’s current use of the most effective method mentioned. Only ~ 1% of the sample report dual use of a hormonal method and a condom. Our measure of current contraceptive use does not capture periodic use of a secondary method

^b^This category includes current users of implants, IUDs, injectables, oral pills, emergency contraceptive and the female condom

Table 3 presents the multivariate multinomial logistic regression results of the association between perceptions of peers’ use of FP and current contraceptive use and choice among sexually experienced young men ages 15–24 years. The results show that young men who perceived that most or all their peers use contraceptives were significantly more likely to use condoms than be non-users of a modern method, compared to those who perceived that few or none of their peers use contraception (RRR = 2.12, p < 0.001). In addition, young men who perceived that all or most of their peers use contraceptives were also more likely to report condom use as compared to reporting current use of another modern method (RRR = 2.13, p = 0.034). Table 3 also shows that young men who were in a dating relationship (i.e., they have a girlfriend) and those who were single as compared to those who were ever married were more likely to use condoms as compared to being a non-user of a modern method (RRR = 3.20, p < 0.001 and RRR = 2.14, p = 0.016, respectively). Similarly, young men who were single, meaning that they did not have a girlfriend, as compared to those who were ever married were more likely to report current use of a male condom as compared to use of another modern method (RRR = 6.90, p = 0.021). Young men with one or more children compared to those with no children were less likely to use a condom than another modern method (RRR = 0.17, p < 0.001) and more likely to report use of another modern method as the current method than be a non-user of a modern method (RRR = 4.89, p = 0.001). No associations were found by age, level of education, or place of residence.

**Table 3:** Multinomial logistic regression results for the association between perceptions of peers’ use of FP and current contraceptive use and choice^a^ among sexually experienced young men ages 15–24 in Kenya (n = 1279)

	Male condom vs. non-user of modern method			All other modern methods^b^ vs. non-user of modern method			Male condom vs. All other modern methods^b^		
	RRR	SE	p-value	RRR	SE	p-value	RRR	SE	p-value
All or most peers use contraception to protect from pregnancy or STI (ref: some/none/don’t know)	2.12	0.26	< 0.001	1.00	0.36	0.992	2.13	0.76	0.034
Age (continuous)	1.01	0.03	0.859	1.08	0.09	0.346	0.93	0.08	0.386
Education (ref: college/vocational school)									
None/some primary	0.71	0.19	0.206	0.98	0.74	0.977	0.73	0.53	0.661
Primary completion	0.86	0.17	0.449	1.41	0.90	0.588	0.61	0.37	0.415
Secondary completion	0.99	0.18	0.961	2.08	1.29	0.239	0.48	0.28	0.205
Relationship status (ref: ever married)									
Dating, have a girlfriend	3.20	1.00	< 0.001	1.37	0.70	0.539	2.33	1.21	0.103
Single, do not have a girlfriend	2.14	0.68	0.016	0.31	0.26	0.156	6.90	5.79	0.021
One or more children (ref: none)	0.85	0.21	0.496	4.89	1.96	< 0.001	0.17	0.08	< 0.001
Reported average monthly earnings (KES) (continuous)	1.00	0.00	0.870	1.00	0.00	0.227	1.00	0.00	0.174
Urban (ref: rural)	0.84	0.13	0.267	0.75	0.26	0.412	1.12	0.40	0.749
2019 Survey wave (ref: 2018)	1.06	0.14	0.668	0.95	0.34	0.884	1.11	0.39	0.761

*RRR* relative risk ratio, *SE* standard error

^a^Current contraceptive use is based on the respondent’s current use of the most effective method mentioned. Only ~ 1% of the sample report dual use of a hormonal method and a condom. Our measure of current contraceptive use does not capture periodic use of a secondary method

^b^This category includes current users of implants, IUDs, injectables, oral pills, emergency contraceptive and the female condom

Table 4 presents the multivariate multinomial logistic regression results for the association between perceptions of peers’ use of contraceptives and current contraceptive use and choice among sexually experienced young women ages 15–24 years. The results show that young women who perceived that all or most of their peers use contraceptives were significantly more likely to report current use of condoms or another modern method as compared to being a non-user of a modern method than those who reported that few or none of their peers used contraception (RRR = 2.59, p < 0.001 and RRR = 1.51, p = 0.020, respectively). Similarly, young women who perceived that all or most of their peers use contraceptives were significantly more likely to currently use condoms as compared to another modern method (RRR = 1.71, p = 0.014). Among this sample of young women, those who were older and who had one or more children were more likely to currently use any other modern method as compared to being a non-user of a modern method (RRR = 1.12, p = 0.005 and RRR = 4.53, p ≤ 0.001, respectively) whereas young women who were dating/had a boyfriend or single/no boyfriend were less likely to use any other modern method compared to being a non-user of a modern method (RRR = 0.46, p ≤ 0.001 and RRR = 0.29, p < 0.001, respectively). Young women who were in a dating relationship as compared to those who were ever married were more likely to use condoms compared to being a non-user of modern methods (RRR = 2.54, p ≤ 0.001) as were women with one or more children (RRR = 0.55, p = 0.004). Young women who were dating/had a boyfriend or single/no boyfriend compared to ever married were more likely to use condoms compared to use of another modern method (RRR = 5.46, p ≤ 0.001 and RRR = 3.85, p < 0.001, respectively) and those with one or more children were less likely to use condoms compared to current use of another modern method (RRR = 0.12, p < 0.001). No associations were found by level of education or place of residence.

**Table 4:** Multinomial logistic regression results for the association between perceptions of peers’ use of FP and current contraceptive use and choice^a^ among sexually experienced young women ages 15–24 in Kenya (n = 1191)

	Male condom vs. non-user of modern method			All other modern methods^b^ vs. non-user of modern method			Male condom vs. All other modern methods^b^		
	RRR	SE	p-value	RRR	SE	p-value	RRR	SE	p-value
All or most peers use of contraception to protect from pregnancy or STI (ref: some/none/don’t know)	2.59	0.48	< 0.001	1.51	0.27	0.020	1.71	0.37	0.014
Age (continuous)	0.96	0.05	0.339	1.12	0.04	0.005	0.85	0.05	0.004
Education (ref: college/vocational school)									
None/some primary	0.52	0.24	0.150	1.22	0.50	0.620	0.42	0.24	0.130
Primary completion	1.05	0.33	0.884	1.19	0.43	0.623	0.88	0.39	0.767
Secondary completion	0.78	0.24	0.410	1.41	0.54	0.360	0.55	0.24	0.176
Relationship status (ref: ever married)									
Dating, have a boyfriend	2.54	0.65	< 0.001	0.46	0.09	< 0.001	5.46	1.56	< 0.001
Single, do not have a boyfriend	1.11	0.33	0.729	0.29	0.07	< 0.001	3.85	1.44	< 0.001
One or more children (ref: none)	0.55	0.11	0.004	4.53	0.97	< 0.001	0.12	0.03	< 0.001
Reported average monthly earnings (KES) (continuous)	1.00	0.00	0.155	1.00	0.00	0.898	1.00	0.00	0.187
Urban (ref: rural)	1.13	0.22	0.525	1.23	0.23	0.253	0.92	0.20	0.697
2019 Survey wave (ref: 2018)	1.23	0.22	0.246	1.23	0.21	0.223	1.00	0.22	0.999

*RRR* relative risk ratio, *SE* standard error

^a^Current contraceptive use is based on the respondent’s current use of the most effective method mentioned. Only ~ 1% of the sample report dual use of a hormonal method and a condom. Our measure of current contraceptive use does not capture periodic use of a secondary method

^b^This category includes current users of implants, IUDs, injectables, oral pills, emergency contraceptive and the female condom

## Discussion

Using data from a survey of sexually experienced young women and men in Kenya, this study builds on previous research about social influences on reproductive behaviors in Kenya [20, 22, 33] and found that perceptions of peers’ use of FP was related to young peoples’ current contraceptive use and choice of method. This is the first study to our knowledge to use household survey data from young women and men in Kenya to explore this topic. Among both sexually experienced young women and young men, the perception that their peers used contraceptives was associated with use of condoms over being a nonuser as well as over use of another modern method of contraception. One distinction between young women and men was that young women were more likely to report current use of any other modern method than nonuse when they perceived their peers used contraceptives but we did not find the same among the sample of sexually experienced young men. The lack of a comparable male finding may reflect the small sample of males who reported current use of a non-condom modern method, potentially due to young men not knowing what method their partner uses. These findings highlight the important role that perceptions of peer behavior can play on a young person’s reproductive behaviors.

Our findings are consistent with studies in Kenya which found that there was a positive relationship between perception of community approval of contraceptives and an individual’s own use of contraception among both women and men of reproductive age [18]; this was also shown qualitatively among adolescent girls [33]. Conversely, a 2020 quantitative study by Shakya and colleagues did not find a significant relationship between the perception of friends’ approval of family planning and use of contraceptives among married adolescent girls in Niger [27], yet these findings may not be relevant for the Kenyan context given significant contextual differences between the two countries including higher overall FP use in Kenya. Interestingly, our findings suggest that there may be normative behavior within peer groups that is supportive of contraceptive use, including use of hormonal methods by sexually experienced young women. This finding is particularly interesting in a context where there are embedded social norms at the community level that restrict young people from engaging in sexual activity [40], yet recent research from an unnamed country in SSA suggests that there may be changing attitudes towards young people using contraception [49]. Our study extends this existing research on social influences on contraceptive use and provides more substantial evidence on this relationship for young women and men in Kenya.

The finding that perception of peers' use of contraceptives was associated with use of male condoms over current use of another modern method for both young men and women reflects a choice between method types which may be related to the dual protection against pregnancy and STI afforded by condoms, both of which might be discussed among young people and their peers. Global evidence from several low- and middle-income countries highlights that women of reproductive age make decisions about their own family planning use based on interactions with their social network, often through direct conversations [17, 37], and are more likely to use the methods their social network members use [31, 32]. Yet evidence from Kenya has shown that men’s social networks may be more influential on men’s contraceptive use than the networks of women on women’s contraceptive use [22]. Further, as shown in Malawi, men may make decisions about contraceptive use based on their observations of their network’s behaviors rather than through direct conversations [37]. For young men, they may not discuss their partner’s use of a hormonal method with their peers either because they are unaware their partner is using a method or due to discomfort discussing their partner’s contraceptive use with others. With nearly one third of our sample reporting current use of condoms alone (rather than in combination with other methods), this is likely the method most discussed among young people for its ability to protect against pregnancy and STI. With the current survey questions, we are unable to know which methods are being discussed among peers.

Our study found that there was a preference for condoms among sexually experienced young men and women. This may be due to several factors. Overall, knowledge of contraceptive methods is high among Kenyan youth, with one survey of unmarried, sexually active Nairobi youth reporting that 98.5% of the sample knew of at least one contraceptive method [15]. High knowledge of condoms among young people may be due, in part, to significant efforts to prevent STIs and HIV through social and behavioral change interventions [50]. Yet, in the sample of Nairobi youth discussed earlier, only about 4 out of 10 could correctly answer questions comparing efficacy between the condom and oral pills or the condom and IUD suggesting knowledge gaps still exist [15]. Targeted messaging about STI and HIV appears to have reached young people, as concerns about STI and HIV transmission were high among the sample of Nairobi youth with 55.8% of unmarried, sexually active young men and 28.8% of unmarried, sexually active young women who said they always use a condom [15]. In addition, young people may face fewer barriers to accessing condoms over other modern methods due to providers restricting access to hormonal methods and a wider range of sources for condoms, including pharmacies and shops, which are frequently more accessible, more convenient, and provide privacy and confidentiality [12, 51–53]. These factors contribute to condoms frequently being an entry point to contraceptive use for young people and may further contribute to normalizing condom use amongst this population [54].

This study has a number of strengths and limitations. Among the strengths is that this study focuses on a nationally representative sample of young people from Kenya, including a sample of young men who are often not included in similar studies. Few studies specifically target young people for data collection because of challenges in receiving approval from ethical review boards to interview young people on sensitive topics, obtaining informed consent or assent, or difficulties identifying an appropriate location for the interview [55]. Moreover, surveys that include young people such as the Demographic and Health Survey rarely attempt to modify survey tools in order to take into consideration appropriate and accurate terminology for young people, a simplified sentence structure on par with reading and comprehension levels of young people and a youth-friendly questionnaire flow [56]. In an effort to address these issues, this study utilized a nonstandard, novel survey tool which was designed to solicit more accurate, honest responses from young people. Building rapport and asking questions in a familiar manner helps to make the respondent more comfortable and is particularly important given the focus of this study on sexual and reproductive health. When comparing estimates of modern contraceptive use for all women ages 15–24 from this study (24% in 2018, 26% in 2019) with other nationally representative household surveys from Kenya, such as the DHS (24% in 2014) or PMA2020 (26% in 2018), the results are nearly identical [9, 12]. This suggests that the nonstandard questions for contraceptive use utilized in this study produce estimates that are comparable to other more commonly collected data sources but the study design also permitted including novel, youth-specific questions that would be challenging to ask in larger demographic surveys.

This study also has some limitations. Many studies that explore social factors and contraceptive use create aggregated community level variables using individual responses to reflect community norms and values [47, 48]. Given the sample size and sampling strategy, there were not enough observations to create community average variables for the questions on perceptions of peers’ use of contraceptives or contraceptive use. In addition, existing literature has shown that the size of an individual’s social network as well as other contextual factors are important to understand the underlying mechanisms of how social factors influence behaviors and how the effects may differ among young women and men [21]. Information on the size of the respondents’ social network and other relevant community-level contextual factors were not collected as part of the secondary data used for this study. In addition, the independent variables only capture perceptions of contraceptive use and do not include more specific mention of methods or types of methods used. There were several key variables, such as the age of the partner and fertility intentions that were not measured and may introduce some residual confounding. Additionally, this analysis is focused on a novel strategy to measure current contraceptive use. As shown above, this approach gives similar aggregate levels of use among young women as found in other national-level surveys [15]. That said, there are also limitations with this approach as we classified those young women or men who report infrequent or occasional use of a method, such as condoms or emergency contraception as non-users; this may represent infrequent sex or infrequent use which we were not able to distinguish with the data available. Notably, condom use as reported in our sample is higher among young women than in the PMA surveys [9]; this means that coding occasional users as non-users may not lead to an underestimation of condom use. In addition, young men may not be aware that their partners are using a hormonal method of contraception and the way the question was asked may have missed their partners’ use of hormonal methods; therefore, use of other modern methods among men may be underreported. Finally, cross-sectional data were used for this analysis which allowed for the examination of associations between variables but does not permit an assessment of the direction of causality. It is possible that given the dynamic nature of young people’s lives, the association we have identified is to some extent also due to reverse causality; meaning that the onset of sexual activity and need for contraceptives lead to creation of peer networks which are similar to the respondent, rather than the other way around.

## Conclusion

In the 2019 Kenya Census, approximately 60% of the population was under 25 years of age [57]. Meeting the contraceptive needs of this growing, young Kenyan population is critical to ensure that young people are able to attain their life goals. Being able to plan and manage fertility is critical during this time period as young people are building themselves—through education, employment, and transitioning to adulthood. Thus, having the means to avoid an unintended pregnancy through information and access to a full range of contraceptive methods is important to support young people’s growth and development. This study highlights the importance of perceptions of peers’ contraceptive behaviors on young women and men’s contraceptive use. Future studies could include collection of social network data among young women and men to better understand dynamics within peer groups and how contraceptive method choice by one network member may affect the choices of others. Additionally, in-depth qualitative data may be useful to better understand how and why peers’ use influences contraceptive method choice. Given the important role that peers play during the adolescent and young adult years, it is pertinent to develop strategies that ensure that young people have accurate and relevant information to support one another’s healthy reproductive health behaviors.

With this in mind, programs should work to disseminate and share information about contraceptive methods broadly through various strategies including social media, mass media, outreach workers, and drama events. Behavior change communication activities that engage young women and men will help to promote positive norms among young people, including males, based on factual information about all available methods. This may include the promotion of positive deviants through these media channels, such as some of the characters in the Shujaaz comic books, which provide entertaining, relatable content to educate the reader while also normalizing positive contraceptive behaviors (https://www.shujaazinc.com/). In addition, youth leaders within communities can be identified to hold discussion groups with other youth to share information about family planning methods. These groups could include younger and older female and male youth who can share experiences about contraceptive methods and which methods may suit young people at different points in the life course. Activities that work to create positive social norms amongst sexually experienced young women and men regarding contraceptive method choice are critical to ensuring that young people are able to avoid unintended pregnancies and plan and manage their fertility.

## Data Availability

Aggregate level de-identified data tables and reports are available at https://www.shujaazinc.com/. Data are available and can be requested from Anastasia Mirzoyants (anastasia.mirzoyants@shujaazinc.com); a formal request will need to be submitted with a data sharing agreement.

## Abbreviations

FP: Family planning
HIV: Human immunodeficiency virus
IUD: Intrauterine device
LMIC: Low- and middle- income countries
SSA: Sub-Saharan Africa
STI: Sexually transmitted infection

## References

[1] Sully EA, Biddlecom A, Darroch JE, et al. Adding It Up: Investing in Sexual and Reproductive Health, 2019. 2020. New York: Guttmacher Institute, https://www.guttmacher.org/report/adding-it-upinvesting-in-sexual-reproductive-health-2019.

[2] Ameyaw EK, Budu E, Sambah F (2019). Prevalence and determinants of unintended pregnancy in sub-Saharan Africa: a multi-country analysis of demographic and health surveys. PLoS ONE.

[3] Yinger N. Policy Brief: meeting the need, fulfilling the promise: youth and long-acting reversible contraceptives. Population Reference Bureau. 2016;(Policy Brief):1–7.

[4] Chandra-Mouli V, Parameshwar PS, Parry M (2017). A never-before oppportunity to strengthen investments and action on adolescent contraception, and what we must do to make full use of it. Reprod Health.

[5] Fikree FF, Lane C, Simon C (2017). Making good on a call to expand method choice for young people: turning rhetoric into reality for addressing Sustainable Development Goal Three. Reprod Health.

[6] United Nations, Department of Economic and Social Affairs, Sustainable Development. Sustainable Development Goals: Goal 3. [cited February 18, 2021]. Available from: https://sdgs.un.org/goals/goal3.

[7] Magadi M, Agwanda AO (2008). Determinants of transitions to first sexual intercourse, marriage and pregnancy among female adolescents: evidence from South Nyanza, Kenya. J Biosocial Sci.

[8] Speizer IS, Guilkey D, Calhoun LM (2017). Examination of youth sexual and reproductive health transitions in Nigeria and Kenya using longitudinal data. BMC Public Health.

[9] PMA2020—Kenya. Adolescents and Young Adults Health Brief, November—December 2018. Website: https://www.pma2020.org/sites/default/files/PMA2020-Kenya-R6-Adolescent-Brief.pdf. Accessed on July 10, 2019.

[10] National Public Radio. As Kenya Keeps Schools Shut, Teen Pregnancies are Rising. [cited February 18, 2021]. Available from: https://www.npr.org/2020/07/11/889718651/as-kenya-keeps-schools-shut-teen-pregnancies-are-rising.

[11] African Institute of Development Policy. Teen pregnancy in Kenya: Verifying the data and the facts. [cited February 18, 2021]. Available from: https://www.afidep.org/news-release-teen-pregnancy-in-kenya-verifying-the-data-and-the-facts/.

[12] Kenya National Bureau of Statistics (KNBS) and ICF Macro. Kenya Demographic and Health Survey 2014. 2015. Calverton, Maryland: KNBS and ICF Macro.

[13] Vargas Nunes Coll C, Ewerling F, Hellwig F (2019). Contraception in adolescence: the influence of parity and marital status on contraceptive use in 73 low- and middle- income countries. Reprod Health.

[14] Radovich E, Dennis ML, Wong KLM (2018). Who meets the contraceptive needs of young women in sub-Saharan Africa?. J Adolesc Health.

[15] International Centre for Reproductive Health-Kenya (ICRHK) & PMA Agile. Nairobi Youth Respondent-Driven Sampling Survey: Final Report. 2019. Performance Monitoring for Action Technical Report. Baltimore, Maryland, USA: Bill & Melinda Gates Institute for Population and Reproductive Health, Johns Hopkins University Bloomberg School of Public Health.

[16] Shujaaz Inc. Cross-sectional survey of youth aged 15–24 in Kenya, April-May 2019 (N=2,020). [cited November 10, 2020]. Available from: https://www.shujaazinc.com/news-publications.

[17] Rutenberg N, Watkins SC (1997). The buzz outside the clinics: conversations and contraception in Nyanza Province, Kenya. Stud Fam Plan.

[18] Dynes M, Stephenson R, Rubardt M, Bartel D (2012). The influence of perceptions of community norms on current contraceptive use among men and women in Ethiopia and Kenya. Health Place.

[19] Lowe SMP, Moore S. Social networks and female reproductive choices in the developing world: a systematized review. Reprod Health. 2014; 11. 10.1186/1742-4755-11-85 PMC4275947 25495334

[20] Kohler HP, Behrman JR, Watkins SC (2001). The density of social networks and fertility decisions: evidence from South Nyanza District, Kenya. Demography.

[21] Boulay M, Valente TW (1999). The relationship of social affiliation and interpersonal discussion to family planning knowledge, attitudes and practice. Int Fam Plan Perspect.

[22] Behrman JR, Kohler H-P, Watkins SC (2002). Social networks and changes in contraceptive use over time: evidence from a longitudinal study in rural Kenya. Demography.

[23] Madhavan S, Adams A, Simon D (2003). Women’s Networks and the social world of fertility behavior. Int Fam Plan Perspect.

[24] Godley J (2001). Kinship networks and contraceptive choice in Nang Rong, Thailand. Int Fam Plan Perspect.

[25] Rimal R, Lapinski MK (2015). A re-explication of social norms, ten years later. Commun Theory.

[26] Costenblader E, Lenzi R, Hershow RB, Ashburn K, McCarraher DR (2017). Measurement of social norms affecting modern contraceptive use: a literature review. Stud Fam Plan.

[27] Shakya HB, Challa S, Nouhou AM, et al. Social network and social normative characteristics of married female adolescents in Dosso, Niger: Associations with modern contraceptive use. Global Public Health. 2020. 10.1080/17441692.2020.1836245 PMC8060360 33091326

[28] Rimal RN, Sripad P, Speizer IS, Calhoun LM. Interpersonal communication as an agent of normative influence: a mixed method study among the urban poor in India. Reprod Health. 2015; 12(71). 10.1186/s12978-015-0061-4 PMC4533786 26265221

[29] Kaggwa EB, Diop N, Storey JD (2008). The role of individual and community normative factors: a multilevel analysis of contraceptive use among women in union in Mali. Int Fam Plan Perspect.

[30] Sedlander E, Rimal RN (2019). Beyond individual-level theorizing in social norms research: how collective norms and media access affect adolescents’ use of contraception. J Adolesc Health.

[31] Valente TW, Watkins SC, Jato MN (1997). Social network associations with contraceptive use among Cameroonian women in voluntary associations. Soc Sci Med.

[32] Gayen K, Raeside R (2010). Social networks and contraception practice of women in rural Bangladesh. Soc Sci Med.

[33] Ochako R, Mbondo M, Aloo S (2015). Barriers to modern contraceptive methods uptake among young women in Kenya: a qualitative study. BMC Public Health.

[34] Ochako R, Temmerman M, Mbondo M, Askew I. Determinants of modern contraceptive use among sexually active men in Kenya. Reprod Health. 2007; 14(56). 10.1186/s12978-017-0316-3 PMC5408470 28449723

[35] Kann Sanchez E, Speizer IS, Tolley E (2020). Influences on seeking a contraceptive method among adolescent women in three cities in Nigeria. Reprod Health.

[36] Mwaisaka J, Gonsalves L, Thiongo M (2020). Exploring contraception myths and misconceptions among young men and women in Kwale County, Kenya. BMC Public Health.

[37] Paz Solden VA (2004). How family planning ideas are spread within social groups in rural Malawi. Stud Fam Plan.

[38] Berndt TJ (1979). Developmental changes in conformity to peers and parents. Dev Psychol.

[39] Steinberg L, Monahan KC (2007). Age differences in resistance to peer influence. Dev Psychol.

[40] Velonjara J, Crouthamel B, O’Malley G (2018). Motherhood increases support for family planning among Kenyan adolescents. Sex Reprod Healthc.

[41] Tavrow P, Karei EM, Obbuyi A, Omollo V (2012). Community norms about youth condom use in western Kenya: is transition occurring?. Afr J Reprod Health.

[42] Harrison A, Smit J, Hoffman S, Nzama T, Leu CS, Mantell J, Stein Z, Exner T (2012). Gender, peer and partner influences on adolescent HIV risk in rural South Africa. Sexual Health.

[43] Giles M, Liddell C, Bydawell M (2005). Condom use in African adolescents: the role of individual and group factors. AIDS Care.

[44] O’Leary A, Jemmott JB, Jemmott LS, Bellamy S, Ngwane Z, Icard L, Gueits L (2012). Moderation and mediation of an effective HIV risk-reduction intervention for South African adolescents. Ann Behav Med.

[45] Eggers SM, Aaro LE, Bos AER, Mathews C, de Vries H (2014). Predicting condom use in South Africa: a test of two integrative models. AIDS Behav.

[46] Sedlander E, Bingenheimer JB, Lahiri S, Thiongo M, Gichangi P, Munar W, Rimal RN (2021). Does the belief that contraceptive use causes infertility actually affect use? Findings from a social network study in Kenya. Stud Fam Plan.

[47] Ahinkorah BO. Predictors of modern contraceptive use among adolescent girls and young women in sub-Saharan Africa: a mixed effects multilevel analysis of data from 29 demographic and health surveys. Contracept Reprod Med. 2020; 5(32). 10.1186/s40834-020-00138-1 PMC7678092 33292675

[48] Mutumba M, Wekesa E, Stephenson R. Community influences on modern contraceptive use among young women in low and middle income countries: a cross-sectional multi-country analysis. BMC Public Health. 2018; 18 (430). 10.1186/s12889-018-5331-y PMC5879615 29609567

[49] Senderowicz L (2019). “I was obligated to accept”: a qualitative exploration of contraceptive coercion. Soc Sci Med.

[50] Michielsen K, Chersich MF, Luchters S (2010). Effectiveness of HIV prevention for youth in sub-Saharan Africa: systematic review and meta-analysis of randomized and nonrandomized trials. AIDS.

[51] Chandra-Mouli V, Akwara E (2020). Improving access to and use of contraception by adolescents: what progress has been made, what lessons have been learnt, and what are the implications for action?. Best Pract Res Clin Obstet Gynaecol.

[52] Tumlinson K, Okigbo C, Speizer I (2015). Provider barriers to family planning access in urban Kenya. Contraception.

[53] Gonsalves L, Wyss K, Cresswell JA (2020). Mixed-methods study on pharmacies as contraception providers to Kenyan young people: who uses them and why?. BMJ Open.

[54] Ouma L, Bozkurt B, Chanley J, Power C, Kakonge R, Adeyemi OC, Kudekallu RJ, Madsen EL (2021). A cross-country qualitative study on contraceptive method mix: contraceptive decisionmaking among youth. Reprod Health.

[55] Bassett R, Beagan BL, Ristovski-Slijepcevic S, Chapman GE (2008). Tough teens: the methological challenges of interviewing teemagers as research participants. J Adolesc Res.

[56] Omrani A, Wakefield-Scurr J, Smith J, Brown N (2019). Survey development for adolescents aged 11–16 years: a developmental science-based guide. Adolesc Res Rev.

[57] Kenya National Bureau of Statistics. 2019 Kenya Population and Housing Census. Volume III: Distribution of Population by Age and Sex. December 2019. Nairobi, Kenya: Kenya National Bureau of Statistics.

